# Biomechanical effects of metastasis in the osteoporotic lumbar spine: A Finite Element Analysis

**DOI:** 10.1186/s12891-018-1953-6

**Published:** 2018-02-05

**Authors:** Giuseppe Salvatore, Alessandra Berton, Hugo Giambini, Mauro Ciuffreda, Pino Florio, Umile Giuseppe Longo, Vincenzo Denaro, Andrew Thoreson, Kai-Nan An

**Affiliations:** 1 Department of Orthopaedic and Trauma Surgery, Campus Bio-Medico University, Via Alvaro del Portillo, 200, 00128 Trigoria, Rome, Italy; 2 Biomechanics Laboratory, Division of Orthopaedic Research, Mayo Clinic, Rochester, MN USA

**Keywords:** Metastasis, Vertebral fracture, Vertebroplasty, Osteoporosis, Finite element analysis, Lumbar spine

## Abstract

**Background:**

Cancer patients are likely to undergo osteoporosis as consequence of hormone manipulation and/or chemotherapy. Little is known about possible increased risk of fracture in this population. The aim of this study was to describe the biomechanical effect of a metastatic lesion in an osteoporotic lumbar spine model.

**Methods:**

A finite element model of two spinal motion segments (L3-L5) was extracted from a previously developed L3-Sacrum model and used to analyze the effect of metastasis size and bone mineral density (BMD) on Vertebral bulge (VB) and Vertebral height (VH). VB and VH represent respectively radial and axial displacement and they have been correlated to burst fracture. A total of 6 scenarios were evaluated combining three metastasis sizes (no metastasis, 15% and 30% of the vertebral body) and two BMD conditions (normal BMD and osteoporosis).

**Results:**

15% metastasis increased VB and VH by 178% and 248%, respectively in normal BMD model; while VB and VH increased by 134% and 174% in osteoporotic model. 30% metastasis increased VB and VH by 88% and 109%, respectively, when compared to 15% metastasis in normal BMD model; while VB and VH increased by 59% and 74% in osteoporotic model.

**Conclusion:**

A metastasis in the osteoporotic lumbar spine always leads to a higher risk of vertebral fracture. This risk increases with the size of the metastasis. Unexpectedly, an increment in metastasis size in the normal BMD spine produces a greater impact on vertebral stability compared to the osteoporotic spine.

## Background

The spine is the most common site for skeletal metastasis, with one third of all cancer patients developing metastases of the spine [1]. Because advancements in oncological treatments have improved patients’ survival, the prevalence of spinal metastases is bound to increase [2]. Vertebral fractures caused by spine metastases result in pain, deformity, loss of mobility, and/or neurological complications, significantly affecting quality of life [3–5].

Many patients with metastases of the spine are likely to decrease their bone mineral density (BMD), leading to osteopenia or osteoporosis, as a consequence of hormone manipulation and/or chemotherapy [6], increasing the risk of vertebral fractures. Snyder et al. developed a computed tomography-based structural analysis (CTRA) method to predict fracture risk associated with osteolytic vertebral lesions [7]. Although highly sensitive and more specific than radiographs, validation studies are still ongoing. On the other hand, little is known about the increased risk of fracture in osteoporotic patients with metastatic lesions. Predictive tools, such as dual absorptiometry (DXA), quantitative computed tomography-based finite element analysis (QCT/FEA), biomechanical computed tomography-based FEA (BCT/FEA), have been implemented to improve fracture risk assessment in osteoporotic patients, but they have not been considered for osteoporotic cancer patients [8–11].

Biomechanical studies investigating the risk of fracture in metastatic spines lack realistic models and are not ideal for parametric analyses [12]. Because cadaveric studies are performed with normal spines, simulated lytic defects are typically developed by removing a core of trabecular bone and penetrating the cortical structure [13–16]. Similarly, clinical studies, including retrospective reviews of patients, can hardly extrapolate the influence of every individual variable as the patients population is generally heterogeneous and uncontrolled multiple factors can influence the results [12]. On the other hand, finite element analysis, successful in predicting failure loads and fracture patterns for bone structures [8, 17–24], allows a parametric representation of complex geometric and material property distributions.

The aim of this study was to evaluate the biomechanical effects of a metastatic lesion in an osteoporotic model of the lumbar spine.

## Methods

### Finite element model

**Fig. 1:**
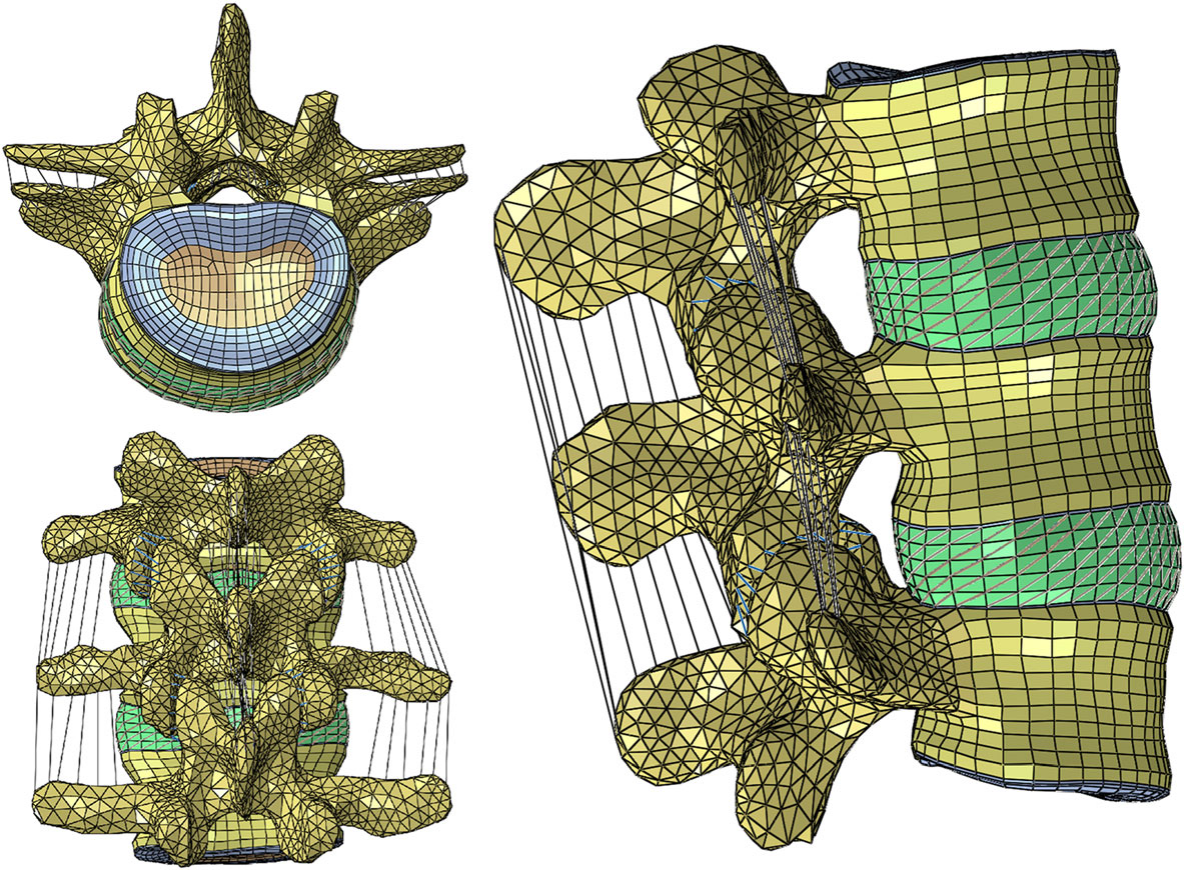
Finite element model of two spinal motion segments (L3-L5)

A finite element model of two spinal motion segments (L3-L5) was extracted from a previously developed and validated three-dimensional, nonlinear, ligamentous L3-Sacrum model [25, 26] (Fig. 1). Development of the model is described in more details in the prior publication [25] and summarized here for convenience. The geometry and dimensions of the model were obtained from a high-resolution computed tomography scan (Siemens Helical CT Scanner, Siemens Corp., Munich, Germany - 0.293 mm in plane pixel size, 0.4 mm slice thickness) of a fresh, frozen human cadaveric spine specimen (male, 52 yrs. old). DICOM images were imported into Mimics image processing and editing software (Materialise US, Plymouth, MI USA) for segmentation. Since soft tissue was poorly visualized on CT scans, the discs were generated using the wrap function in 3-matic 4.2 (Materialise, Ann Arbor, MI, USA). Discs was modeled as a composite of a solid matrix with embedded fibers in concentric rings. The Nucleus pulposum was surrounded by seven annulus fibrosus lamellae, containing two evenly spaced layers of fibers oriented at approximately ±30° to the horizontal plane [27, 28]. The fibers were defined using 3-D two-node truss elements. Collagenous fiber content varies from 23% in the outermost layer to 5% in the innermost layer [29, 30]. Thus, the cross-sectional area of the truss elements was determined based on the annular layer volume and the number of elements to satisfy the fiber content distribution. The fiber thickness and stiffness increased in the radial direction. A “no compression” option was defined for the annulus fibrosus such that the elements resist tension only. The solid matrix of the annulus fibrosus was modeled as a neo-Hookean solid, while the nucleus pulposum was linearly elastic.

The finite element mesh was generated using HyperMesh 10.0 (Altair Engineering, Inc., Troy, MI, USA).

The model included the vertebral bodies consisting of a cortical shell, cancellous centrum, endplates and posterior elements. Intervertebral discs consisted of a nucleus pulposus, annulus ground substances, and annulus fibrosus (seven layers). All major ligaments were represented: (anterior longitudinal, posterior longitudinal, intertransverse, ligamentum flavum, interspinous, supraspinous, and capsular). The ligaments were modeled with 3-D two node truss elements and assigned nonlinear hypoelastic material properties, which allow axial stiffness to be a function of axial strain. At initially low strains the ligaments exhibit low stiffness, but as the strains increase the ligament stiffness increases. The cross-sectional areas of each ligament were obtained from literature. All elements representing the ligaments were aligned in the anatomical fiber direction and unstressed at the unloaded state [28].

The metastasis was represented as a lytic bone lesion. Material properties for all structures are summarized in Table 1.

**Table 1:** Finite element model material properties

	Elastic Modulus (MPa)	Poisson’s Ratio
Vertebra		
Cancellous Bone	100	0.2
Cortical Bone	12,000	0.3
Vertebral Bony Endplate	4000	0.3
Cartilage Endplate	5	0.17
Posterior Bone	3500	0.25
Intervertebral Disc		
Nucleus Polposus	1	0.49
Annular Fibers	Neo-Hooke	
Annular Layers	Neo-Hooke	
Joint		
Facet Joints	3500	
Ligaments		
Anterior Longitudinal	15.6–20.0	0.3
Posterior Longitudinal	10.0–20.0	0.3
Intertransverse	12–58.7	0.3
Ligamentum Flavum	13.0–19.5	0.3
Interspinous	9.8–12.0	0.3
Supraspinous	8.8–15.0	0.3
Capsular	7.5–33.0	0.3
Metastasis		
Lytic Bone Metastasis	0.01	0.4995

### Boundary conditions and loads

Nodes at the lower surface of the L5 vertebra, corresponding to the endplate, were encastred and constrained from all three axes of rotation and translations. A reference node was placed 10 mm above the L3 vertebra and surface nodes from the vertebra and the reference node were selected to form rigid bodies and create a kinematic coupling. This constrain limited the motion of aforementioned nodes to the motion applied to the reference node [31]. An axial compressive load of 1200 N was applied to the superior reference node atop the L3 vertebra to represent upper body weight. This load corresponds to a compressive force on the lumbar spine for an individual standing upright holding an 8.3 kg mass with outstretched arms [32].

### Parametric analyses

**Fig. 2:**
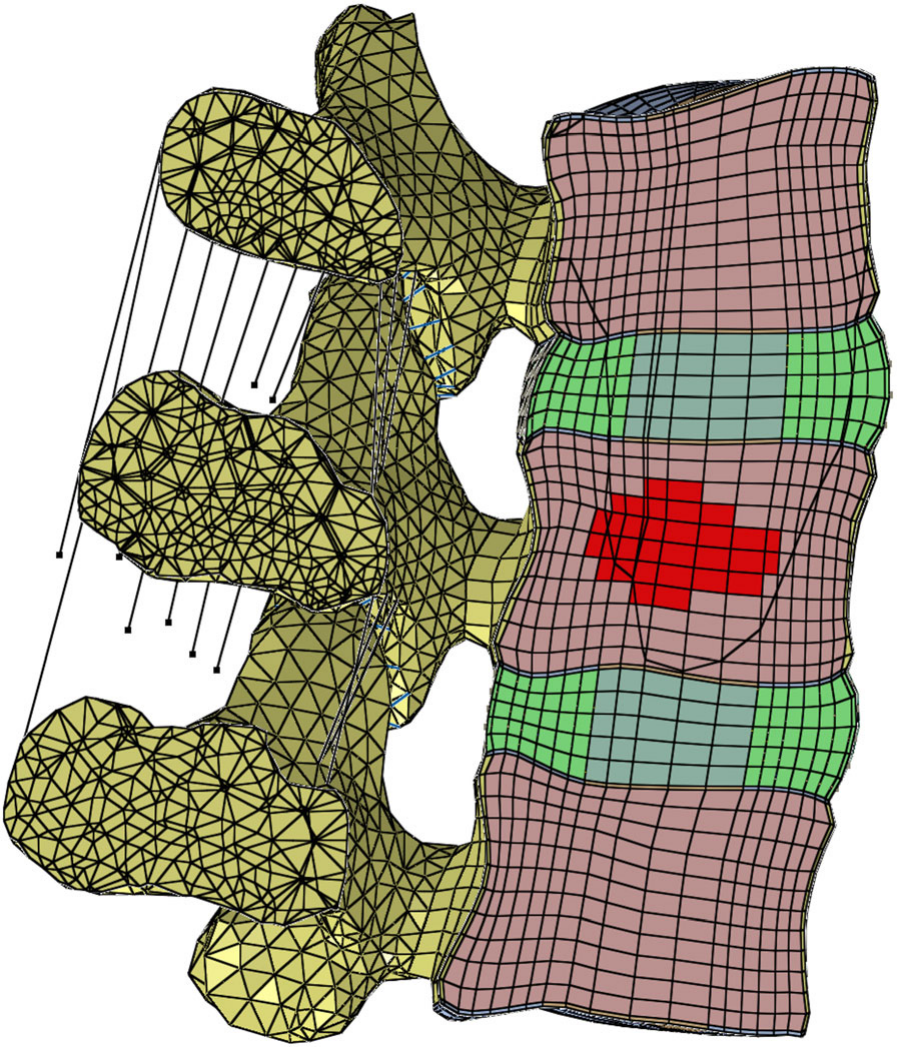
The metastatic lesion was configured as roughly elliptical mass by selecting a core of elements that covered approximately 15% of the volume of L4 vertebral body

A parametric analysis was performed varying metastasis size and BMD. Metastatic lesion was varied to represent 15% and 30% of the vertebral body volume by selecting elements at the central core of the L4 vertebral body. (Fig. 2). Osteoporosis was defined by a 66% reduction in the elastic moduli of all bony structures for the cancellous bone, and by 33% for the cortical shell, the endplates, and the posterior elements [33]. Soft tissue structures were left unchanged. A total of 6 scenarios were evaluated, as described in Table 2. Scenario 1 (Normal BMD - Metastasis size 0%) was used as a baseline for comparison with other normal BMD conditions. Scenario 4 (Osteoporotic - Metastasis size 0%) was used as baseline for comparison with other osteoporotic conditions.

**Table 2:** Investigated scenarios

Scenario	BMD	Metastasis Size
1	Normal	0%
2	Normal	15%
3	Normal	30%
4	Osteoporotic	0%
5	Osteoporotic	15%
6	Osteoporotic	30%

Scenario 1 (Normal BMD - Metastasis size 0%) and scenario 4 (Osteoporotic - Metastasis size 0%) were used as baseline

### Outcomes variables

Vertebral fracture risk was analyzed based on vertebral bulge (VB) and vertebral height (VH) outcomes [13]. Vertebral bulge, representing the maximum radial displacement at the transverse midline of the vertebral body, has been noted to correlate with load-induced spinal canal narrowing, vertebral cortex tensile hoop strains, and bone marrow pressurization [13]. Vertebral height, representing the maximum axial displacement of the endplates, characterizes the risk of endplate failure leading to subsequent burst fracture [13].

**Fig. 3:**
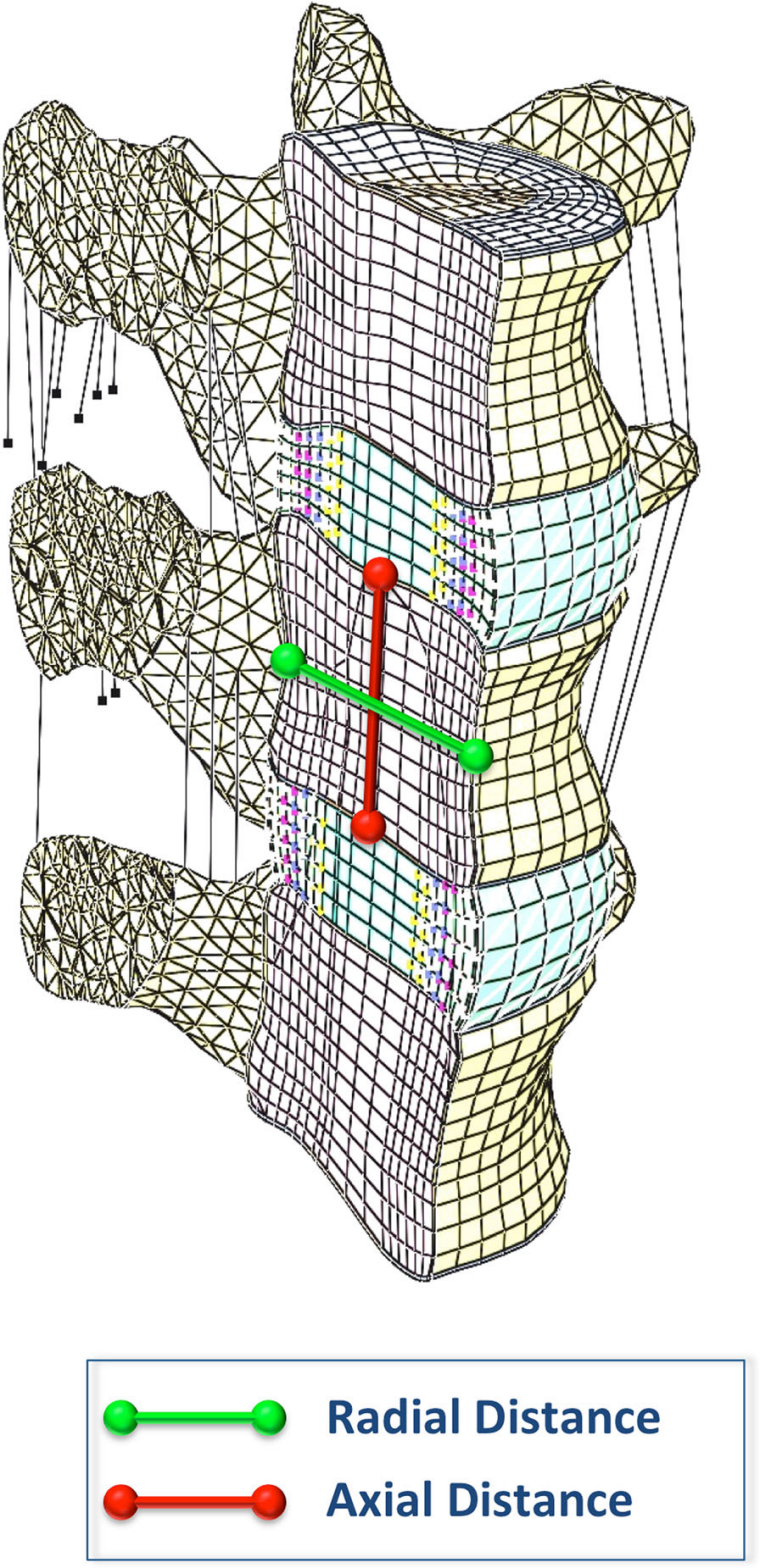
Radial Distance (green line): distance between two standard nodes at the mid-height of L4 on the sagittal plane. Axial Distance (red line): distance between two standard nodes at the center of the inferior and superior endplates of L4

The distance between two standard nodes at the mid-height of L4 on the sagittal plane was measured (Radial Distance, Fig. 3). VB was calculated as the difference between the Radial Distance before and after the axial compressive load was applied (VB = Radial Distance after load – Radial Distance before load).

The distance between two standard nodes at the center of the inferior and superior endplates of L4 was measured (Axial Distance, Fig. 3). VH was calculated as the difference between the Axial Distance before and after the axial compressive load was applied (VH = Axial Distance before load – Axial Distance after load).

## Results

**Fig. 4:**
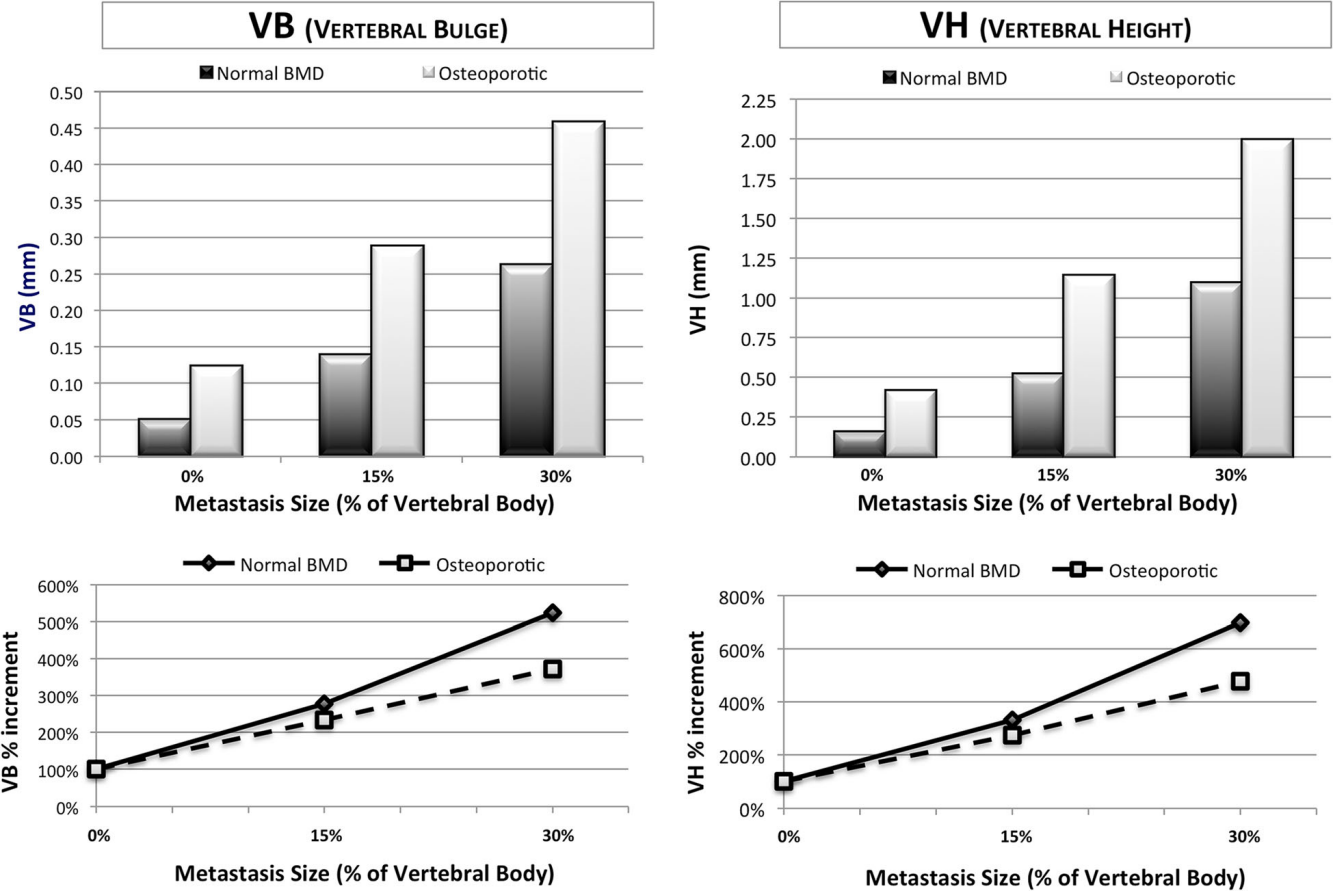
Histograms show values of VB and VH (in mm) in simulated scenarios. Results show that osteoporotic models are less stable compared to respective normal BMD models. Scatterplots show increments of VB and VH (in percentage) normalized to the respective baseline values, to highlight the trend of increment. Results show that metastasis size greater affects normal BMD models stability compared to osteoporotic ones

Results for the simulations with metastasis were normalized to relative baseline scenarios of intact vertebra without metastasis (Scenario 1: VB 0.05 mm, VH 0.15 mm and Scenario 4: VB 0.123 mm, VH 0.417 mm). In normal BMD scenarios, 15% metastasis increased VB by 178% (0.139 mm) and VH by 248% (0.522 mm); while a 30% metastasis increased VB by 424% (0.262 mm) and VH by 626% (1.09 mm). A comparison of metastatic lesion size showed a 30% metastasis to increase VB by 88% (0.139 mm vs 0.262 mm) and VH by 109% (0.522 vs 1.09) when compared to a 15% metastasis. In osteoporotic scenarios, a 15% metastasis increased VB by 134% (0.288 mm) and VH by 174% (1.145 mm); while a 30% metastasis increased VB by 272% (0.458 mm) and VH by 479% (1.996 mm). Comparing the two metastatic scenarios (15% and 30%), VB increased by 59% (0.288 vs 0.458) and VH by 74% (1.145 vs 1.996). When same metastasis size scenarios were compared, the osteoporotic spine had larger VB and VH values compared to normal BMD scenario. Results are summarized in Table 3 and Fig. 4.

**Table 3:** VB and VH for each scenario, absolute values

BMD	Normal		Osteoporotic	
Metastasis size	15%	30%	15%	30%
VB	0.133	0.262	0.288	0.458
VH	0.522	1.09	1.145	1.996

## Discussion

The purpose of this study was to describe the biomechanical effect of a metastatic lesion in an osteoporotic lumbar spine model in order to better understand the risk of vertebral fractures in this population. A finite element model of two spinal motion segments (L3-L5) was used to analyze the effect of metastasis size and osteoporosis on VB and VH. Results from the study showed osteoporosis can represent a risk of fracture regardless of metastasis size compared to patients with normal BMD. Furthermore, an increase in metastasis size has a greater impact on the risk of fracture in patients with normal BMD compared to osteoporotic patients.

A previous study by Taneichi et al. [34] identified the following criteria of impending collapse: 1) 50–60% involvement of the vertebral body with no destruction of other structures, or 25–30% involvement with costovertebral joint destruction in the thoracic spine; and 2) 35–40% involvement of vertebral body, or 20–25% involvement with posterior elements destruction in thoracolumbar and lumbar spine. It is well known that the load bearing capacity of bone is influenced by the geometry, location, biological activity of the tumor, and the geometry and material properties of the host bone [34]. Several studies have investigated the risk of vertebral fracture in osteoporotic bones [35–45]. However, there is still a lack of knowledge relating the interactive and/or cumulative effect of metastatic cancer and osteoporosis [46, 47].

To the best of our knowledge, this is the first three-dimensional, anatomical model of two spinal motion segments (L3-L5) that investigates a metastatic lesion in an osteoporotic spine. A previous study by Whyne et al., investigated the effects of tumor size, material properties and compressive loading rate on vertebral strength, using a two-dimensional, symmetric finite element model of the L1 vertebral body without posterior elements [48]. Two additional studies by Whyne et al. implemented a three-dimensional finite element model of the L1 vertebra including the posterior arch but no additional posterior elements were represented [13, 49]. These studies showed tumor size to be the predominant contributor towards the risk of initiating a burst fracture, followed by the applied load magnitude and bone density. However, the biomechanical response, including stress distribution and geometrical changes, is more complex in a spine segment comprised of the vertebral bodies with posterior elements and soft tissues (intervertebral discs and ligamentous structures). The posterior elements, facet joints and ligaments share a substantial portion of the loads applied to the spine, stabilizing and preventing vertebral bulge [50]. Tschirat et al. developed a geometrical three-dimensional finite element model of a thoracic spine segment to understand the effects of vertebral level, geometry, and metastasis on the cortical shell [51, 52]. The study demonstrated that upper thoracic vertebrae are at greater risk of burst fracture, and that kyphotic segments, ribcage and transcortical tumor provided lower risk of burst fracture initiation [52]. Moreover, the effect of multiple loading conditions on metastatically-involved thoracic spinal motion segment was investigated showing axial loading as the predominant load type leading to increased risk of burst fracture initiation [51]. However, the load distribution in the lumbar spine might differ from that observed in the thoracic spine due to the presence of the ribcage, vertebral size, lordotic angle, and articular facet angles. Our results showed osteoporosis to highly affect vertebral outcomes of the model. Previous studies have only modified trabecular bone material properties based on an assumed apparent density [13, 48, 49]. In order to obtain a reliable representation of an osteoporotic spine, the current model included changes in the material properties of the cortical shell, endplates and posterior elements.

This study has limitations. The L3-L5 finite element model was extracted from a previously developed and validated three-dimensional, nonlinear, ligamentous L3-Sacrum model [25, 26]. Prior validation of the L3-Sacrum model allows considering results derived from the L3-L5 model as reasonable. However, the two spinal motion segment model cannot be considered as properly validated. The metastatic lesion was represented as an ellipsoid, and tumor shape can influence vertebral bulge and vertebral axial displacements [33, 41]. However, the ellipsoidal geometry is frequently used in finite element models of metastasis [13, 41, 49]. Second, only an axial compressive load of 1200 N was studied. However, this represents a compressive force on the lumbar spine for an individual standing upright holding an 8.3 kg mass with outstretched arms [32]. Lower loading regime should be studied in order to simulate daily life tasks of endstage cancer patients that can be translated to clinical practice. Third, additional motions or loads were not simulated. Tschirhart et al. [51] suggested focusing primarily on axial compressive loading rather than complex load/boundary conditions since it is the predominant load type leading to increased risk of burst fracture initiation of the thoracic spine and it is likely to be the same for the lumbar segment. Fourth, it could be argued that metastatic lesions are more common in the thoracic spine rather than in the lumbar spine. However, we aimed to evaluate the effect of axial loading without the influences of the ribs, which can contribute to reduce the effective axial loading applied on the vertebra. Therefore, we decided to study the lumbar spine segment. In future studies, we are planning to study localization of metastasis to the thoracic spine. Fifth, we only simulated a lytic metastasis. Blastic lesions are frequent and should be investigated in future studies. However, it should be taken into account that, both lytic and blastic metastasis lead to a decrease in bone mineralization [46, 53]. Mineral content have been demonstrated to be strongly correlated with strength/stiffness [54–56]. Thus, the reason for fracture of a metastatic vertebra is related to poor bone quality, both in case of lytic and blastic metastasis. Finally, our parametric study was limited to metastasis size and bone mineral density. Future studies should evaluate metastasis location, vertebral level, pedicle involvement, metastasis type, and disc degeneration.

## Conclusions

A metastasis in the osteoporotic lumbar spine always leads to a higher risk of vertebral fracture. This risk increases with the size of the metastasis. Unexpectedly, an increment in metastasis size in the normal BMD spine produces a greater impact on vertebral stability compared to the osteoporotic spine.

## Abbreviations

BCT/FEA: Biomechanical computed tomography-based FEA
BMD: Bone mineral density
CTRA: Computed tomography-based structural analysis
DXA: Dual absorptiometry
QCT/FEA: Quantitative computed tomography-based finite element analysis
VB: Vertebral bulge
VH: Vertebral height
